# Multiplex quantitative PCR for single-reaction genetically modified (GM) plant detection and identification of false-positive GM plants linked to *Cauliflower mosaic virus* (CaMV) infection

**DOI:** 10.1186/s12896-019-0571-1

**Published:** 2019-11-07

**Authors:** Aurélie Bak, Joanne B. Emerson

**Affiliations:** Department of Plant Pathology, University of California, Davis, CA 95616 USA

**Keywords:** *Cauliflower mosaic virus*, CaMV, GMO, GM plant, Multiplex qPCR, Detection methods

## Abstract

**Background:**

Most genetically modified (GM) plants contain a promoter, P35S, from the plant virus, *Cauliflower mosaic virus* (CaMV), and many have a terminator, TNOS, derived from the bacterium, *Agrobacterium tumefaciens.* Assays designed to detect GM plants often target the P35S and/or TNOS DNA sequences. However, because the P35S promoter is derived from CaMV, these detection assays can yield false-positives from non-GM plants infected by this naturally-occurring virus.

**Results:**

Here we report the development of an assay designed to distinguish CaMV-infected plants from GM plants in a single multiplexed quantitative PCR (qPCR) reaction. Following initial testing and optimization via PCR and singleplex-to-multiplex qPCR on both plasmid and plant DNA, TaqMan qPCR probes with different fluorescence wavelengths were designed to target actin (a positive-control plant gene), P35S, P3 (a CaMV-specific gene), and TNOS. We tested the specificity of our quadruplex qPCR assay using different DNA extracts from organic watercress and both organic and GM canola, all with and without CaMV infection, and by using commercial and industrial samples. The limit of detection (LOD) of each target was determined to be 1% for actin, 0.001% for P35S, and 0.01% for both P3 and TNOS.

**Conclusions:**

This assay was able to distinguish CaMV-infected plants from GM plants in a single multiplexed qPCR reaction for all samples tested in this study, suggesting that this protocol is broadly applicable and readily transferrable to any interested parties with a qPCR platform.

## Background

A genetically modified (GM) plant possesses genetic material that has been modified in order to introduce a new gene or trait to the plant, for example herbicide resistance, disease resistance, or insect tolerance [1]. Most of the engineered genetic constructs in GM plants are built with the 35S promoter (P35S) from *Cauliflower Mosaic Virus* (CaMV) and the NOS terminator (TNOS) derived from the soil-borne bacterium, *Agrobacterium tumefaciens.* Indeed, according to studies published in 2014–2015, P35S and TNOS are used in 65.7 and 53.49% of all commercialized GM crops, respectively, and either or both were used in 81.4% of all GM events, indicating that most GM plants can be detected by methods that target P35S and TNOS [2, 3]. To comply with GM crop legislation and respect consumer transparency, reliable GM plant detection methods have been developed, mostly focused on P35S and TNOS [4–8] (Bak A, Emerson JB. Towards distinguishing Cauliflower mosaic virus (CaMV) infection from genetic modification (GM) in crop plants: detection assays and biology, management, and food safety of CaMV. Submitted). However, it is known that CaMV infection of non-GM plants can yield false-positive results from GM plant detection assays, due to the presence of the P35S region in both the CaMV genome and many GM crops [9, 10]. Therefore, a non-GM plant infected with CaMV could be incorrectly identified as a genetically modified organism (GMO).

Most GM plant detection methods follow DNA-based approaches, particularly polymerase chain reaction (PCR) and quantitative PCR (qPCR), because they allow for fast and specific GM plant screening [2, 11]. The PCR method uses DNA sequence-specific primers and a DNA polymerase enzyme to amplify new DNA strands from existing DNA strands (e.g., DNA from the sample of interest) as templates [12]. qPCR methods use, in addition to the same PCR reagents, a double-stranded deoxyribonucleic acid (dsDNA) fluorescent binding dye or a DNA sequence-specific fluorescent probe that allows for the quantification of the newly amplified DNA molecules by measuring the fluorescence produced during each PCR cycle [13, 14]. The number of amplification cycles required to reach a fixed fluorescence signal threshold (i.e., exceeding background levels to be considered detected) is called the Ct (cycle threshold) value. Two different methods of qPCR are extensively used, SYBR Green (non-specific dsDNA binding) and TaqMan [13, 15]. The TaqMan method uses a DNA sequence-specific hydrolysis probe labelled with a fluorophore and a quencher [16], and although the Taqman method is more specific than SYBR Green, both of these assays can be adapted to be relatively or absolutely quantitative [17–19].

New highly sensitive, reliable, and cost-effective GM plant detection methods continue to be developed [6, 20–22]. Nevertheless, few methods can discriminate between GM plants and CaMV infection, and all currently available methods would require multiple assays to identify false-positive GM plants. For example, in [23], the authors described PCR tests to detect P35S and the P3 gene of CaMV for differentiating between GM plants and CaMV infection in parallel reactions [23]. Here we sought to adapt these single-reaction PCR assays, together with PCR assays for TNOS, into a single multiplex qPCR assay to detect CaMV infection and GM plants that use constructs with P35S or TNOS, thereby minimizing reagent costs and processing time.

In this paper, we present an absolute multiplex TaqMan qPCR assay combining primers and TaqMan probes that can identify most GM events and can discriminate GM plants from CaMV infection. Four TaqMan qPCR probes with different fluorescence wavelengths were designed to target actin (a positive-control gene universal to plants), P35S, P3 (a CaMV specific gene), and TNOS. After the validation of the primers and probes using regular PCR and SYBR Green methods, the TaqMan method was used and multiplexed for the simultaneous detection of all four targets. The specificity of the primers and probe sets was tested using different combinations of plant DNA (non-GM and GM plants, infected or not with CaMV, and commercial and industrial samples) and the limit of detection (LOD) for each of the four targets was assessed. We present both the optimization process for developing this assay, along with tests to assess its robustness. For all samples tested, this assay was able to distinguish CaMV-infected plants from GM plants in a single multiplexed qPCR reaction.

## Results

**Fig. 1:**
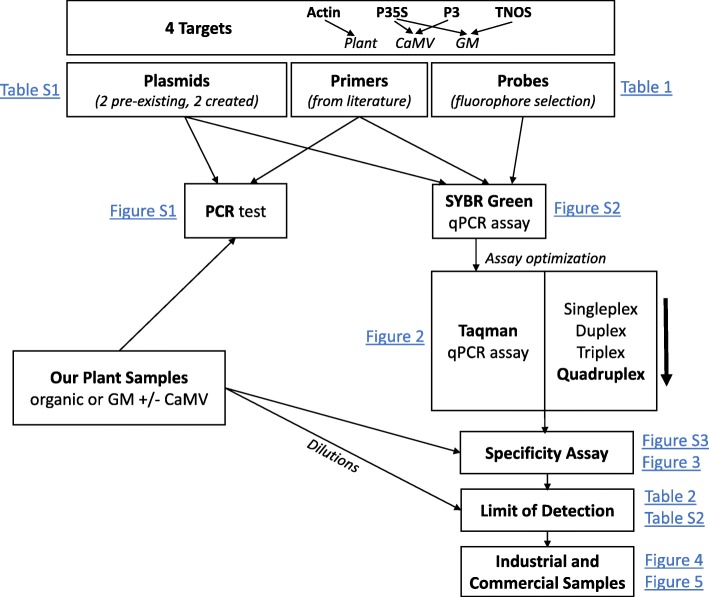
Workflow chart. Diagram representing the different steps followed to develop the multiplex qPCR assay

**Fig. 2:**
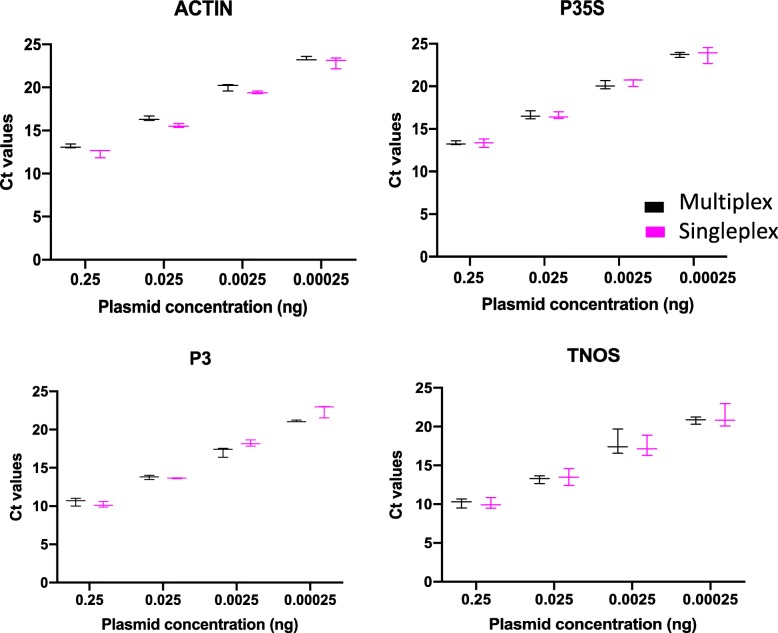
Multiplex to singleplex qPCR Ct comparison. Graphics showing the comparison of the Ct values for each target (actin, P35S, P3, and TNOS) obtained from the multiplex assay (in black) and from the singleplex assay (in pink). The error bars show results from three independent experiments. (*N* = 3, no significant differences between any pair of multiplex vs. singleplex Ct values for the same plasmid concentration and target, Student’s t test, *P* < 0.05)

**Fig. 3:**
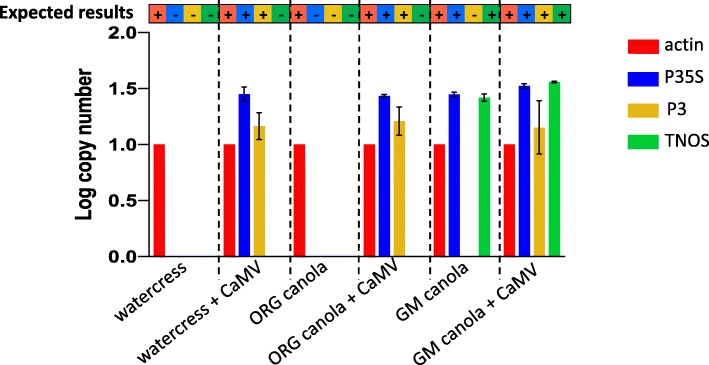
Specificity of the multiplex qPCR assay. Log copy number of each target (actin, P35S, P3, and TNOS) for organic (watercress and ORG Canola) or GM (GM canola) plants infected or not with CaMV. The expected results are presented in the bar above the graphic (+ indicates expected amplification, − indicates no expected amplification). The error bars show results from three independent experiments, and no error bars are shown for actin because all copy numbers were normalized to actin

**Fig. 4:**
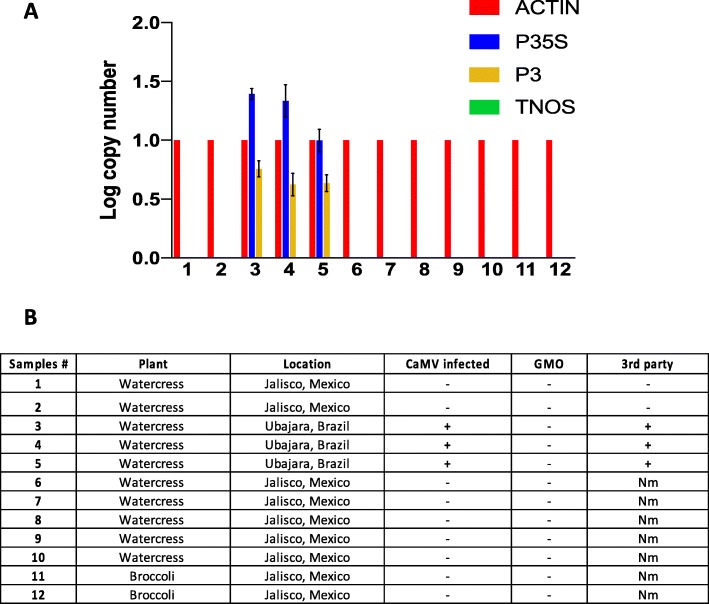
Multiplex qPCR assay on industrial samples. **a** Log copy number of each target (actin, P35S, P3, and TNOS) for different plant samples provided by Nutrilite by Amway. The error bars show results from three independent experiments, and no error bars are shown for actin because all copy numbers were normalized to actin. **b** Sample information for results in (a), + (detected) or – (not detected) indicates the result of our multiplex assay. The final column, “3rd party,” has GM plant detection results (+/−) from an independent 3rd party assay that could not distinguish CaMV infection from GM plant detection. GMO = genetically modified organism, Nm = not measured

**Fig. 5:**
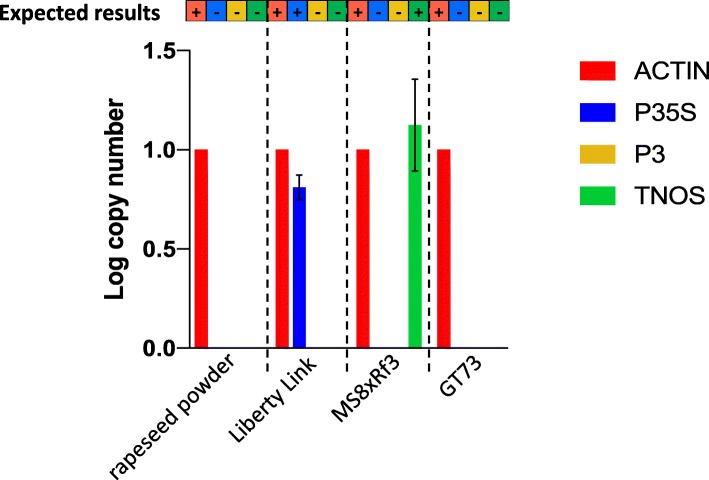
Multiplex qPCR assay on commercially available samples. Log copy number of each target (actin, P35S, P3, and TNOS) for different purchased plant and/or DNA samples. The expected results are presented in the bar above the graphic (+ indicates expected amplification, − indicates no expected amplification). Liberty Link has P35S, MS8xRf3 has TNOS, and GT73 has neither P35S nor TNOS. The error bars show results from three independent experiments, and no error bars are shown for actin because all copy numbers were normalized to actin

A workflow chart is presented in Fig. 1, showing the steps that we followed to develop the TaqMan multiplex qPCR assay for distinguishing true GM plants from false-positive GM plants due to CaMV infection. Briefly, literature-derived forward and reverse specific primer sets (Table 1) [23–25] were tested by PCR to confirm that they specifically amplified the corresponding target and template (Additional file 2: Figure S1). One primer from each set was then tested via qPCR with the probe as the second primer to ensure probe specificity, using a SYBR Green assay (Additional file 3: Figure S2). The forward and reverse primer pairs were then tested together with the fluorescent probe for each target, using a TaqMan assay, initially in singleplex (one target per reaction) and then progressively increasing the number of targets and probes by multiplexing two, three, and, eventually, all four sets in the same reaction. The efficacy of the TaqMan quadruplex (hereafter, multiplex) assay with all four targets (actin, P35S, P3, and TNOS) was confirmed by comparison of each target in TaqMan singleplex vs. multiplex qPCR assays (Fig. 2). After validation, the specificity of the multiplex assay was tested using different combinations of plant DNA (Fig. 3) and commercial and industrial plant DNA samples (Fig. 4 and Fig. 5). Finally, the limit of detection (LOD) was determined for each target (Table 2 and Additional file 4: Table S2). The following sections describe further details for each step of this process.

**Table 1:** Primer table. Table with the primers and probes used in the qPCR assays

Target	Primers	Sequences	Amplicon (bp)	References
Actin = Plant reference gene	Actin	Forward 5′-CAAGCAGCATGAAGATCAAGGT-3’	103	[24]
		Reverse 5′-CACATCTGTTGGAAAGTGCTGAG-3’		
		Probe 5′-HEX-CCTCCAATCCAGACACTGTACTTYCTCTC-BHQ-3’		
CaMV Promoter 35S	P35S	Forward 5′-CGTCTACAAAGCAAGTGGATTG-3’	79	[25]
		Reverse 5′-TCTTGCGAAGGATAGTGGGATT-3’		
		Probe 5′-FAM-TCTCCACTGACGTAAGGGATGACGCA-QSY-3′		
CaMV gene P3	P3	Forward 5′-TGAAATCCTCAGTGACCAAAAATC-3′	152	[23]
		Reverse 5′-TACAAGGACAATCATTGATGAGC-3′		
		Probe 5′-ABY-AAGCCGTTGCAGCGAAAATCGTTAATGA-QSY-3’		
A.tumefaciens nopaline synthase terminator	TNOS	Forward 5′-GTCTTGCGATGATTATCATATAATTTCTG-3’	151	[24]
		Reverse 5′-CGCTATATTTTGTTTTCTATCGCGT-3’		
		Probe 5′-JUN-AGATGGGTTTTTATGATTAGAGTCCCGCAA-QSY-3′

**Table 2:** Limit of Detection (LOD) of the multiplex qPCR assay. The LOD for each target (actin, P35S, P3, and TNOS) is the percentage of plant DNA containing the target of interest diluted in relevant background DNA (i.e., background not containing the target of interest, see Methods) at the lowest concentration in which the target was detectable; 10 samples were tested for each dilution, and in each case, all 10 were positive (detected) at the LOD

	LOD (%)
actin	1
P35S	0.001
P3	0.01
TNOS	0.01

### Confirmation of primer and plasmid efficacy by PCR

Forward and reverse specific primers (see Table 1) were tested by regular PCR to confirm amplification of the corresponding target DNA sequence, using plasmid and then plant DNA as templates (Additional file 2: Figure S1). After cloning the actin and P3 genes into separate pDonr207 vectors, two resulting plasmids (#1 and #2) for each target were tested by PCR using the actin and P3 primers, respectively. The PCR positively amplified the corresponding targets, which appeared as single gel electrophoretic bands of the appropriate size, with no observed non-specific amplification (Additional file 2: Figure S1a). The actin and P3 plasmids labeled #1 were then used for subsequent experiments. P35S and TNOS primers were similarly able to specifically amplify their targets in the pICH51277 and pICH41421 plasmids, respectively (Additional file 2: Figure S1a).

After primer efficacy was confirmed using the plasmid DNA templates, primers were similarly tested using plant DNA templates. The actin primers were confirmed by PCR using all three of our uninfected plant DNA extracts (GM canola, watercress, and non-GM canola) separately as templates (Additional file 2: Figure S1b). The efficacy of P35S and TNOS primers was confirmed by PCR using GM canola DNA as template (Additional file 2: Figure S1c). Finally, the CaMV infection success in all of our infected plants and our ability to specifically amplify CaMV targets in DNA from these CaMV-infected plants was confirmed by PCR using P3 and P35S primers on template DNA from CaMV-infected watercress, non-GM canola, and GM canola (Additional file 2: Figure S1d).

### Confirmation of primer and probe efficacy using SYBR green qPCR assays

Forward and reverse specific primers for each target were used to confirm amplification of the target, using a SYBR Green assay and the corresponding plasmid as template DNA. Subsequently, one primer from each pair was replaced with the probe (without the fluorescent dye) for that target and used with the second primer (see Table 1) in a SYBR Green assay to ensure that the probe sequences were effective prior to purchasing probes with fluorophores (Additional file 3: Figure S2). For both of these tests, we generated melting curves to track double-stranded DNA dissociation with rising temperature in the SYBR Green qPCR reaction, leading to an increase in the fluorescence absorbance intensity. The temperature at which 50% of the DNA is denatured is known as the melting temperature, and this is manifested as a peak in the melting curve. The SYBR Green method allowed us to generate a melting curve for each primer set, including both forward-reverse and primer-probe pairs, which allowed us to ensure that each primer set yielded a specific PCR product (one peak at one temperature) without non-specific amplification or primer-dimers, either of which would have resulted in peaks at different temperatures [26]. Indeed, we confirmed the specificity of all of our primer and probe sets with the formation of a single peak in the melting curve (Additional file 3: Figure S2).

### Development and validation of the multiplex qPCR assay

The fluorophores added to the probes were chosen to minimize spectral profile overlap so that each amplified target would emit fluorescence in a different wavelength, allowing the four targets to be detected in the same qPCR reaction. The four chosen fluorophores were FAM™, HEX®, ABY® and JUN®, with emission spectra peaks at 517 nm, 551 nm, 580 nm, and 617 nm, respectively. To develop the TaqMan multiplex qPCR assay, a plasmid dilution series was tested by TaqMan qPCR. All four targets were first tested in singleplex reactions, then a duplex reaction with actin plus P35S was tested, followed by a triplex reaction with actin, P35S and P3 before the quadruplex reaction with all four targets (actin, P35S, P3, and TNOS). The primer and probe concentrations of the reaction mix were optimized at each step for the best amplification of the four targets (see Methods for the final, optimized protocol).

Once the multiplex protocol was established, the efficacy of the multiplexed assay was validated using a plasmid dilution series tested in singleplex (reactions containing a single set of primers and the probe for one specific target) with Ct values compared to Ct values for the same target in multiplex (reactions containing primer and probe sets for all four of the targets) (Fig. 2) [27, 28]. No significant difference in sensitivity or efficiency was found between the singleplex and multiplex qPCRs for any of the four targets (*P* < 0.05). Indeed, the Ct values for each target in the multiplex reaction were very similar to those obtained from the singleplex reaction (< 1 difference in the Ct values).

### Primer efficiency and generating standard curves for the multiplex qPCR assay

A serial dilution of the four plasmids mixed together in UltraPure water was used to determine the primer efficiency and generate standard curves. The amplification efficiencies for each of the four targets ranged between 92 and 96%, which is well within the acceptable range of 80 to 120% (the ideal amplification efficiency is 100%, assuming that the PCR product concentration doubles every cycle during the exponential phase of amplification [29, 30]). For each multiplex qPCR assay, a standard curve for each primer set was generated, which could be used for placement of unknown targets to determine their log copy numbers and Ct values to assess whether or not a given target was detected. The Y-intercept, slope, and R^2^ data for the standard curves associated with the primer efficiency tests are summarized in Additional file 4: Figure S3.

Most qPCR assays use standard curves based on plasmid dilution in pure water, as we did above, and therefore, we used plasmid dilutions for all of the standard curves and multiplex qPCR measurements reported here. However, we recognize that most actual targets from samples of interest will be amplified from a “noisy” plant DNA background (as in, the target sequence will be within the plant genome, as opposed to in a relatively small plasmid), so we were curious to test the difference in amplification efficiency when plasmids were diluted in relevant DNA background instead of just water. To test this, a serial dilution of the four plasmids mixed together and diluted in plant DNA (for P35S, TNOS, and P3) or bacterial DNA (for actin) was used in multiplex qPCR assays to see how much the primer efficiency differed from results from plasmids in water. The amplification efficiencies for each of the four targets was between 80 and 89% when the plasmids were diluted in background DNA, which was 7–13% lower than in pure water but still within the acceptable amplification efficiency range of 80–120% (Additional file 4: Figure S3). As dilution in organismal DNA (here, plant or bacterial DNA, as opposed to plasmid DNA) is a non-standard approach, we did not use these methods for any of our downstream calculations.

### Specificity of the multiplex qPCR assay

Our different plant DNA samples (GM and non-GM plants with or without CaMV infection) were used to test the specificity of the multiplex qPCR assay. We wanted to ensure that all expected targets amplified and no non-specific amplification was observed. For these measurements, primer efficiencies were adjusted to 100% for the calculation of the log copy number, which was normalized to the reference target, actin (see Methods). All expected targets amplified, and no cross-reactions were observed between the different targets in the multiplex qPCR assay (Fig. 3). Specifically, TNOS did not amplify in any non-GM samples, CaMV P3 did not amplify in any uninfected plants, and P35S did not amplify in any uninfected, non-GM plants. As expected, actin amplified well from all plant DNA samples, TNOS amplified well from GM plant DNA, P3 amplified well from CaMV-infected plant DNA, and P35S amplified well from all tested GM and/or CaMV-infected plant DNA. These results indicate that the multiplex qPCR assay has 100% specificity.

### Sensitivity (limit of detection) of the multiplex qPCR assay

In order to determine the limit of detection (LOD) for the P35S, P3, and TNOS targets via the multiplex qPCR method, different concentrations of CaMV-infected GM canola DNA (which would be positive for P35S, P3, and TNOS) were diluted in uninfected, non-GM canola DNA (i.e., a complex plant DNA background that did not contain P35S, P3, or TNOS). The LOD of actin was determined using different concentrations of plant DNA diluted in bacterial DNA. Based on the specificity of the multiplex assay results, the Ct value 28 was the threshold Ct considered to be positive. Ten replicates were used for each assay, and the LOD of P3 and TNOS was found to be 0.01%, the LOD of P35S was 0.001%, and the LOD of actin was 1% (Table 2). These percentages represent the concentration of the target that was detectable within the background DNA and are approximately the concentration of target expected to be detectable by this method. The LOD of actin, which appears to be very high compared to the LOD of P3, P35S, and TNOS, is probably due to the poor efficacy of the actin dye-quencher combination (the least sensitive of the four fluorescent dyes was assigned to the actin probe specifically because sensitivity to the actin control was the least important, relative to sensitivity to P3, P35S, and TNOS targets). Similarly, differences in the LOD of the other targets are likely due to differences in the efficacy of the dye-quencher combination for each target.

To evaluate the sensitivity of detection of CaMV infection in a GM plant DNA background, the Ct values obtained for each target (actin, P35S, P3, and TNOS) for different percent dilutions (10, 5, 2, 1, 0.1 and 0.01%) of CaMV-infected GM plant DNA in uninfected GM plant DNA were evaluated and compared to the Ct values obtained from uninfected GM plant DNA alone (Additional file 5: Table S2). As shown in Additional file 5: Table S2, the Ct values of the different targets changed only slightly between the different concentrations of CaMV-infected GM plant DNA tested, suggesting that the assay is not effective at determining specific CaMV concentrations in a GM plant background. However, CaMV was detected (via the amplification of P3) in a GM plant background even at extremely low concentrations of CaMV infection, suggesting that the assay is highly sensitive to CaMV infection (Additional file 5: Table S2). Together, these results mean that the multiplex qPCR assay described here can be used to detect CaMV infection with high sensitivity but should not be used to quantify the amount of infection.

### Applicability to test samples

The multiplex qPCR assay was tested on different plant samples provided by Nutrilite by Amway (Fig. 4). Initially, we (the study authors) were blind to the expected results from each of these samples, and the results from prior testing of the same samples by a third party were provided to us after we generated our results in Fig. 4. We were informed that the tested samples were broccoli and watercress from organic (and non-GM plant-containing) farms in Brazil and Mexico (Fig. 4b) and that three samples (numbers 3, 4, and 5) had previously tested positive for GM plants by a proprietary third-party assay. We were informed that false-positive GM plant detection due to CaMV infection was strongly suspected for these three samples (and none of the others), but that CaMV infection had never been previously confirmed or disambiguated from GM plant detection. In our multiplex qPCR assay, those three plant samples (3, 4, and 5) tested positive for CaMV infection (positive for P3 and P35S) but negative for GM plants (no amplification of TNOS) (Fig. 4a). Though proprietary, we infer that the third-party GM plant detection assay was based on detection of P35S, which would be present in a CaMV-infected non-GM plant, and therefore that the third-party assay could not disambiguate GM plant detection from a false-positive GM plant due to CaMV infection. Our addition of P3 (which was positive for these three samples, indicating CaMV infection) and TNOS (which was negative for these three samples, indicating that a GM plant was not likely, though our assay does not consider all possible terminators) allowed us to disambiguate a very likely false-positive GM plant due to CaMV infection. All of the remaining samples were GM plant- and CaMV-negative (no amplification of P35S, TNOS, or P3), consistent with the organic source of the samples and indicative of a lack of detectable CaMV infection (Fig. 4a).

The multiplex qPCR assay was also tested on commercial samples purchased from Sigma-Aldrich (GMO Genomic DNA Standard Set, Cat. No 55231) to further ensure the specificity of the assay. The canola DNA LIBERTY LINK™ Falcon GS40/90, MS8xRf3, and GT73 ROUNDUP READY™ samples gave the expected results using the multiplex qPCR assay, specifically, amplification of P35S for LIBERTY LINK™ Falcon GS40/90, amplification of TNOS for MS8xRf3, and no amplification of TNOS or P35S for GT73 ROUNDUP READY™ (Fig. 5). A non-GM plant, non-CaMV-infected rapeseed (also called canola) powder was also used as a negative control after DNA extraction (Rapeseed, Cat. # ERMBF434A - ERM® certified Reference Material, nominal 0% GMO), and as expected, only actin amplified from that sample (Fig. 5).

## Discussion

Here we have described a method that allows, in a single reaction, for the detection of a very low level of CaMV infection (0.01%) in both GM and non-GM plants. The ability of this assay to detect CaMV infection is due to the detection or non-detection of the CaMV-specific gene, P3. Although the assay can clearly show when even a trace amount of CaMV infection is present, it cannot be used to quantify the amount of CaMV infection. The method also allows for the detection of GM plants that contain P35S and/or TNOS, accounting for most known GM plants. Nevertheless, the method cannot detect GMOs that do not use at least one of these two markers. This multiplex qPCR assay can distinguish between CaMV infection and most common GM plants, as follows: detection of P35S and/or TNOS but not P3 would indicate an uninfected GM plant, detection of TNOS (regardless of the other results) would indicate a GM plant, and detection of P35S and P3 but not TNOS would indicate CaMV infection of a likely non-GM plant. There is still the potential for false-positive GM plant detection in this assay, due to potential *Agrobacterium tumefaciens* infection, which could be overcome by considering non-GMO targets in the *A. tumefaciens* genome, as discussed elsewhere [9] (Bak A, Emerson JB. Towards distinguishing Cauliflower mosaic virus (CaMV) infection from genetic modification (GM) in crop plants: detection assays and biology, management, and food safety of CaMV. Submitted). Also, the efficacy of the P3 and P35S primers on divergent CaMV strains is to be determined. For example, a recent in silico test designed to evaluate the specificity of common CaMV primers to a wide diversity of CaMV strains detected 100% sequence homology for the P3 primers used here to 54% (forward) and 78% (reverse) of 96 tested CaMV strains, with the number and locations of SNPs variable among strains [10]. Although these P3 primers were expected to perform the best among the pre-existing primers in that study, primers that target a more diverse range of CaMV strains could be considered for future improvements to this assay, for example, newly designed CaMV ORFV primers with three degenerate base-pairs per primer to target all 96 of the tested strains [10]. Finally, the positive control actin primers should theoretically result in positive PCR products for all plants [24]; this was the case for all plants in our study, but we did not attempt validation of the actin primers beyond the species studied here.

## Conclusions

PCR and qPCR methods have remained the primary GM plant detection techniques. However, most GM plant detection methods do not allow for discrimination between CaMV infection and GM plants, and to our knowledge, there is no currently available technique that allows for rapid, efficient, and relatively affordable disambiguation between CaMV infection and GM plant detection. Here we have described an optimized multiplex qPCR assay for GM plant detection that allows for the detection of most GM plants, along with the identification of false-positive GM plants linked to *Cauliflower mosaic virus* (CaMV) infection in a single reaction. This method should be compatible with and relatively easily transferrable to any diagnostic (or other laboratory) facility with a qPCR machine.

## Methods

### Plant material

Non-GM canola (*Brassica napus*) seeds and non-GM watercress (*Nasturtium officinale*) seeds were purchased from Amazon (www.amazon.com). GM canola (*Brassica napus* cv. HyCLASS 969 Roundup-Ready® which has P35S and TNOS) seeds were provided by Stephen Kaffka (UC Davis). The plants were grown in a greenhouse at 20–25 °C with 14 h light period.

### CaMV infection

Dried turnip tissue infected with wild-type CaMV strain W260 was obtained from James E. Schoelz at the University of Missouri. To prepare inoculum (virus sap) for our plants, dried CaMV-infected turnip tissue was ground in two volumes of 20 mM phosphate buffer (pH 7.2). One-week-old canola and watercress plants were mechanically inoculated with the virus sap and used for experiments at 3 weeks post infection. Two leaves per plant were dusted with carborundum (Sigma-Aldrich, St Louis, MO) to facilitate penetration and rub-inoculated with the virus sap using a cotton-stick, as previously described [31, 32]. Three weeks post infection, two plants from each variety were combined together and their DNA was extracted.

### Plant DNA extraction

For all plant DNA extractions (including those from our own fresh plant tissues, from dried tissue received either as coarse cut or fine ground from Nutrilite by Amway, or from powder (Rapeseed, Cat. # ERMBF434A - ERM® certified Reference Material, nominal 0% GMO)), plant tissues were ground in liquid nitrogen prior to DNA extraction. Plant DNA was then extracted using the DNeasy plant mini kit (Qiagen) following the manufacturer’s recommendations. Following the measurement of the DNA concentration using the Thermo Scientific™ NanoDrop™ OneC Microvolume UV-Vis spectrophotometer, DNA extracts were diluted to a final concentration of 10 ng/μL in nuclease-free water. DNA was stored at − 20 °C until further use.

### Plasmid DNA

Two control plasmids, one containing a single copy of the P35S promoter and the other containing a single copy of the TNOS transcriptional terminator (plasmids pICH51277 and pICH41421, respectively) were provided by Gitta Coaker (UC Davis). Two more control plasmids, one for actin and one for P3 (a CaMV-specific gene), were constructed using the Gateway® cloning technology, according to the manufacturer’s instructions (Invitrogen, Carlsbad, CA, USA), including the design and synthesis of Gateway primers containing the attB and attP sites (Additional file 1: Table S1, att sites added to P3 primers from ref. [23] and actin primers from ref. [24]) to facilitate insertion into the vector. The actin genetic region was amplified by PCR, using DNA extracted from turnip leaves (see method above) as the template and the gene-specific gateway primers (Gtw-Actin) listed in Additional file 1: Table S1. The P3 CaMV gene was amplified by PCR using CaMV-infected turnip DNA (extracted from infected leaves as described above) as the template and the gene-specific gateway primers (Gtw-P3) listed in Additional file 1: Table S1. The PCR products were then inserted into the entry vector pDONR207 via Gateway® BP recombinant reaction between attB and attP sites. After transformation in *Escherichia coli* DH5alpha, the clones were selected on LB agar plates supplemented with gentamycin (50 μg/ml). The plasmids were purified using the Zyppy™ plasmid miniprep kit according to the manufacturer’s recommendations. Finally, the plasmid DNA concentrations were measured using the Thermo Scientific™ NanoDrop™ OneC Microvolume UV-Vis spectrophotometer and diluted to 10 ng/μL in nuclease-free water. Two plasmids (derived from different bacterial colonies) were tested by PCR (Additional file 2: Figure S1) and the plasmids labeled #1 were used for further experiments.

DNA from each of the four plasmids (containing actin, P35S, P3, and TNOS, respectively) was serially diluted at different concentrations (0.25 ng/μL; 0.025 ng/μL; 0.0025 ng/μL and 0.00025 ng/μL), and, depending on the experiment, mixed together (multiplex assay) or used separately (singleplex assay). DNA was stored at − 20 °C until further use.

### Oligonucleotides and probes

Primers and TaqMan® probes used in this experiment are presented in Table 1. The primers were produced by Sigma-Aldrich, and the TaqMan® probes were synthesized by either Sigma-Aldrich (MilliporeSigma, Life Science business of Merck KGaA, Darmstadt, Germany) for HEX®-Actin and FAM™-P35S or Applied Biosystems (Thermo Fisher Scientific, Waltham, Massachusetts, U.S) for ABY®-P3 and JUN®-TNOS. The primers and probes used in this paper were selected because they are widely cited in the literature and known to work on various types of plants and samples in singleplex PCR and/or qPCR reactions [23–25]. The four fluorophores (FAM™, HEX®, JUN®, and ABY®) for the TaqMan probes were selected to minimize overlap of their spectral profiles.

### PCR settings

PCR was performed using forward and reverse specific primers (Table 1) and GoTaq (Promega, Madison, WI, USA) according to the manufacturer’s instructions and using the following program for 35 cycles: denaturation at 95 °C for 1 min, annealing at 55 °C for 1 min, and extension at 72 °C for 12 s. The PCR reaction was then loaded on a 1.5% agarose gel, separated by gel electrophoresis, and visualized with a UV transilluminator to identify PCR products.

### SYBR green qPCR method

SYBR Green qPCR was tested using each plasmid DNA as template with the specific primer set for each target. The qPCR was performed on a QuantStudio 6 Flex instrument (Thermo Fisher Scientific) equipped with a 384-well block. The reaction consisted of a 10 μL amplification mix containing 5 μL of PowerUP™ SYBR™ Green Master Mix (Thermo Fisher Scientific), 400 nM of each primer, and 4 μL of plasmids (at different concentrations: 0.25 ng/μL, 0.025 ng/μL, 0.0025 ng/μL, and 0.00025 ng/μL). No template controls (NTCs) were used as negative controls. All reactions were tested in triplicate. The reactions were performed in standard mode. They consisted of a first step at 50 °C for 2 min, followed by a step at 95 °C for 10 min, followed by 40 cycles of a step at 95 °C for 15 s and a step at 55 °C for 1 min. The fluorescent signals and the melting curves were analyzed using QuantStudio Real-Time PCR software version 1.1 (Thermo Fisher Scientific) with a manual threshold.

### TaqMan qPCR

TaqMan singleplex and multiplex qPCR runs were performed on a QuantStudio 6 Flex instrument (Thermo Fisher Scientific) equipped with a 384-well block. Each multiplex reaction consisted of a 15 μL amplification mix containing 7.5 μL of Multiplex Master Mix (Thermo Fisher Scientific), 400 nM of each primer, 250 nM of FAM™-P35S probe and 300 nM of HEX®-Actin, ABY®-P3 and JUN®-TNOS probes, and 4 μL of sample DNA (40 ng) or 4 μL of plasmids (at different concentrations: 0.25 ng/μL, 0.025 ng/μL, 0.0025 ng/μL, and 0.00025 ng/μL). Singleplex reactions were the same but only included one set of primers and one probe, adjusting the final volume to 15 μL using UltraPure water. Mustang Purple™ dye was used as a passive reference for normalization. No template controls (NTCs) were used as negative controls. All reactions were tested in triplicate. The distribution of samples and controls was not randomized within each PCR plate, but samples and controls were in multiple blocks throughout each plate, meaning that groups of control wells were interspersed with groups of sample wells, and vice versa.

The reactions were performed in standard mode. They consisted of a first step at 50 °C for 2 min, followed by 95 °C for 10 min, followed by 40 cycles of: 95 °C for 15 s then 55 °C for 1 min. The fluorescent signals were analyzed using QuantStudio Real-Time PCR software version 1.1 (Thermo Fisher Scientific) with a manual threshold. Ct values below 28 were considered positive [33].

### Specificity trial

To evaluate the specificity of this multiplex assay, high percentages (≥1% (w/w)) of non-GM and GM plant materials, infected or not infected with CaMV, were tested in triplicate [18]. Specifically, the six different samples tested were: non-GM watercress (there are currently no widely produced GM watercress plants), CaMV-infected non-GM watercress, non-GM canola, CaMV-infected non-GM canola, GM canola, and CaMV-infected GM canola. The assay was considered specific when it amplified all targets expected to be in the sample and no targets not expected to be in the sample.

### Sensitivity trial

To determine the limit of detection (LOD) of the different targets, 10-fold serial dilutions from 1% down to 0.0001% were tested for each target. To obtain these low concentrations of DNA and to test the LOD of P3, P35S, and TNOS, CaMV-infected GM canola DNA was serially diluted in non-GM Canola DNA. Similarly, to test the LOD of actin, plant DNA was serially diluted in bacterial DNA [34, 35].

### Copy number calculation

For absolute quantification, standard curves were obtained for each target based on a dilution series of all four of the DNA plasmids containing the target genes mixed together. The slopes of the corresponding standard curves were used to calculate the amplification efficiency percentage of each primer set using the mathematical formula: 10^(− 1/slope of the standard curve)^-1. The DNA concentrations from the standard curve were converted to the number of copies, using the following formula:

Number of copies = (DNA amount (g) *6022*10^23^) / (plasmid length (bp)*660) where 6022*10^23^ is Avogadro’s number and 660 is the average weight of a base pair (Kamau, 2013).

The Ct values of each target obtained from the multiplex qPCR assays of the plant samples were interpolated as unknowns from the linear regression standard curves to determine the log copy numbers, using the following formula: (Ct – intercept)/slope. The log copy numbers were then adjusted to a 100% primer efficiency, depending on the primer set percentage efficiency from the standard curve. Finally, the log copy numbers were normalized with the actin reference gene, using the following formula: log copy number of the target/log copy number of actin [24].

### Statistical analyses

The statistical analyses were performed using GraphPad Prism version 8.00 for Mac (GraphPad Software, La Jolla California USA, www.graphpad.com) and data were analyzed by Student’s t-tests. All experiments were repeated at least three times, with the number of repeats indicated in the text and/or figure(s).

## Supplementary information

**Additional file 1: Table S1.** Gateway primers used for cloning actin and P3 (Gtw-Actin and Gtw-P3).

**Additional file 2: Figure S1.** Primer sets tested by PCR. a Specific primers tested by PCR using the corresponding plasmids as template. After the cloning of the actin and P3 amplification products, two different plasmids (1 and 2) were tested. b Actin primers tested by PCR using plant DNA extract as template (GM Canola, Watercress and Non-GM Canola). c P35S and TNOS specific primers tested by PCR using GM Canola DNA as template. d P3 and TNOS specific primers tested by PCR on CaMV-infected plants (GM Canola, Non-GM Canola and Watercress).

**Additional file 3: Figure S2.** Melting curves for each primer set and probe obtained by SYBR Green assay. Specific primers were tested by SYBR Green qPCR using the corresponding plasmid as template. Melting curves for each set of primers (Forward + Reverse) or using one Primer + the Probe are shown with the temperature on the x-axis and the derivative reporter (△Rn) on the y-axis. The derivative reporter is calculated as the negative first derivative of the normalized fluorescence (Rn) generated by the reporter during PCR amplification. It allows for visualization of the maximum rate of change in fluorescence during the temperature ramp.

**Additional file 4: Figure S3.** Standard curves for each target. A serial dilution of the four plasmids mixed together and diluted in water (blue) or in plant DNA (green) for P35S, P3, and TNOS or in bacterial DNA (green) for actin were tested in multiplex qPCR to determine primer efficiency and standard curves for each primer set.

**Additional file 5: Table S2.** CaMV-infected GM plant DNA versus uninfected GM plant DNA. Ct values for each target (actin, P35S, P3, and TNOS) for different dilution percentages (10, 5, 2, 1, 0.1 and 0.01%) of CaMV-infected GM plant DNA in GM plant DNA, compared to the Ct values obtained from GM plant DNA alone. Here, GMO is short for “GM plant.”

## Data Availability

The datasets used and/or analyzed during the current study are available from the corresponding author on reasonable request.

## Abbreviations

CaMV: Cauliflower mosaic virus
Ct: Cycle threshold
GM: Genetically Modified
GMO: Genetically Modified Organism
LOD: Limit of detection
NTC: No Template Control
P35S: Cauliflower mosaic virus 35S promoter
PCR: Polymerase Chain Reaction
qPCR: quantitative Polymerase Chain Reaction
TNOS: Nopaline synthase terminator
