# Supplementary figures and images for: Multiplex quantitative PCR for single-reaction genetically modified (GM) plant detection and identification of false-positive GM plants linked to Cauliflower mosaic virus (CaMV) infection
Source: BMC Biotechnol. 2019 Nov 7;19:73. doi: 10.1186/s12896-019-0571-1 (PMC6836441; doi:10.1186/s12896-019-0571-1)

## Slide 1
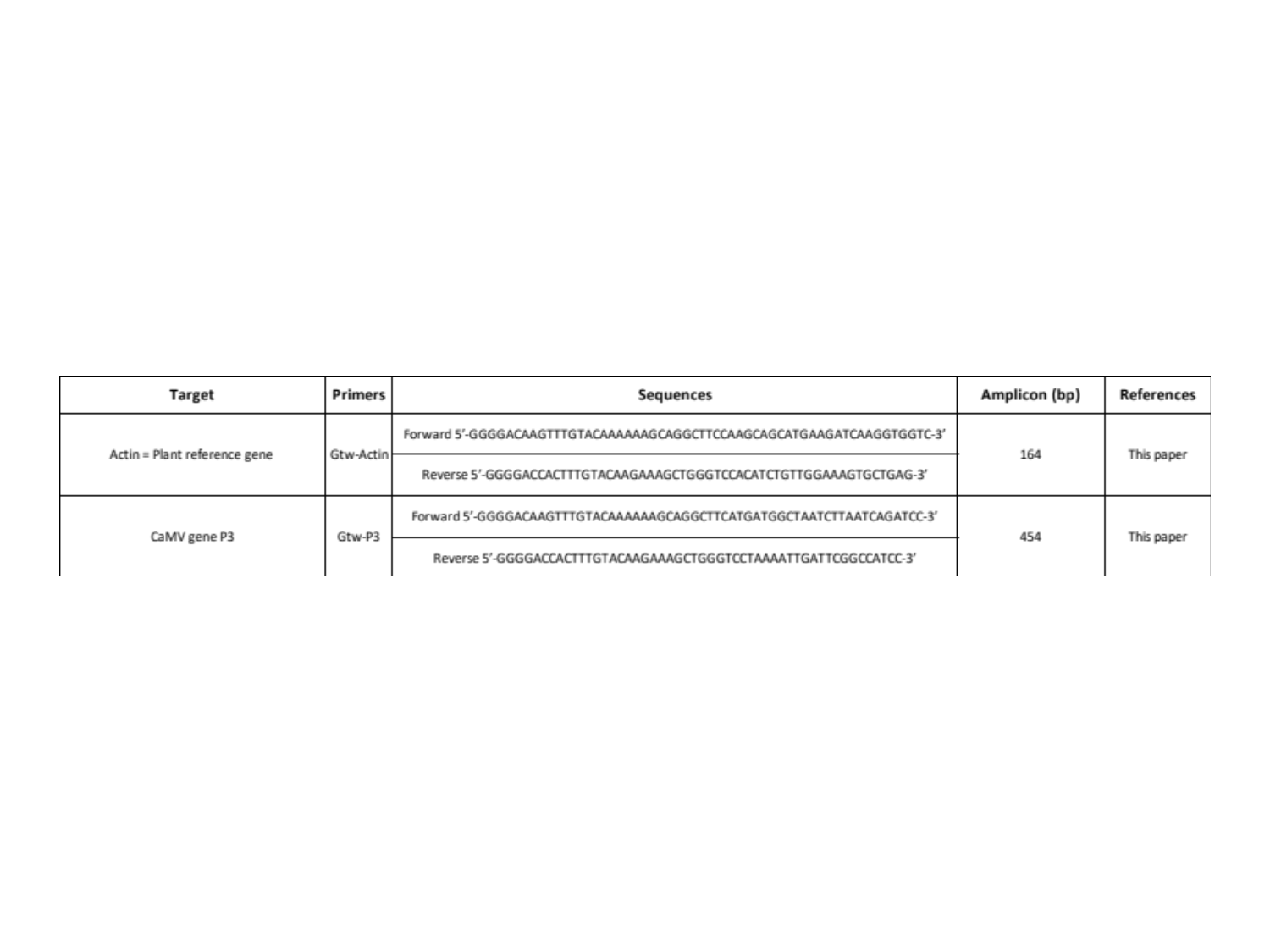

Supplement: Supplementary file 1 — Additional file 1: Table S1. Gateway primers used for cloning actin and P3 (Gtw-Actin and Gtw-P3). [file 12896_2019_571_MOESM1_ESM.pptx]

## Slide 1
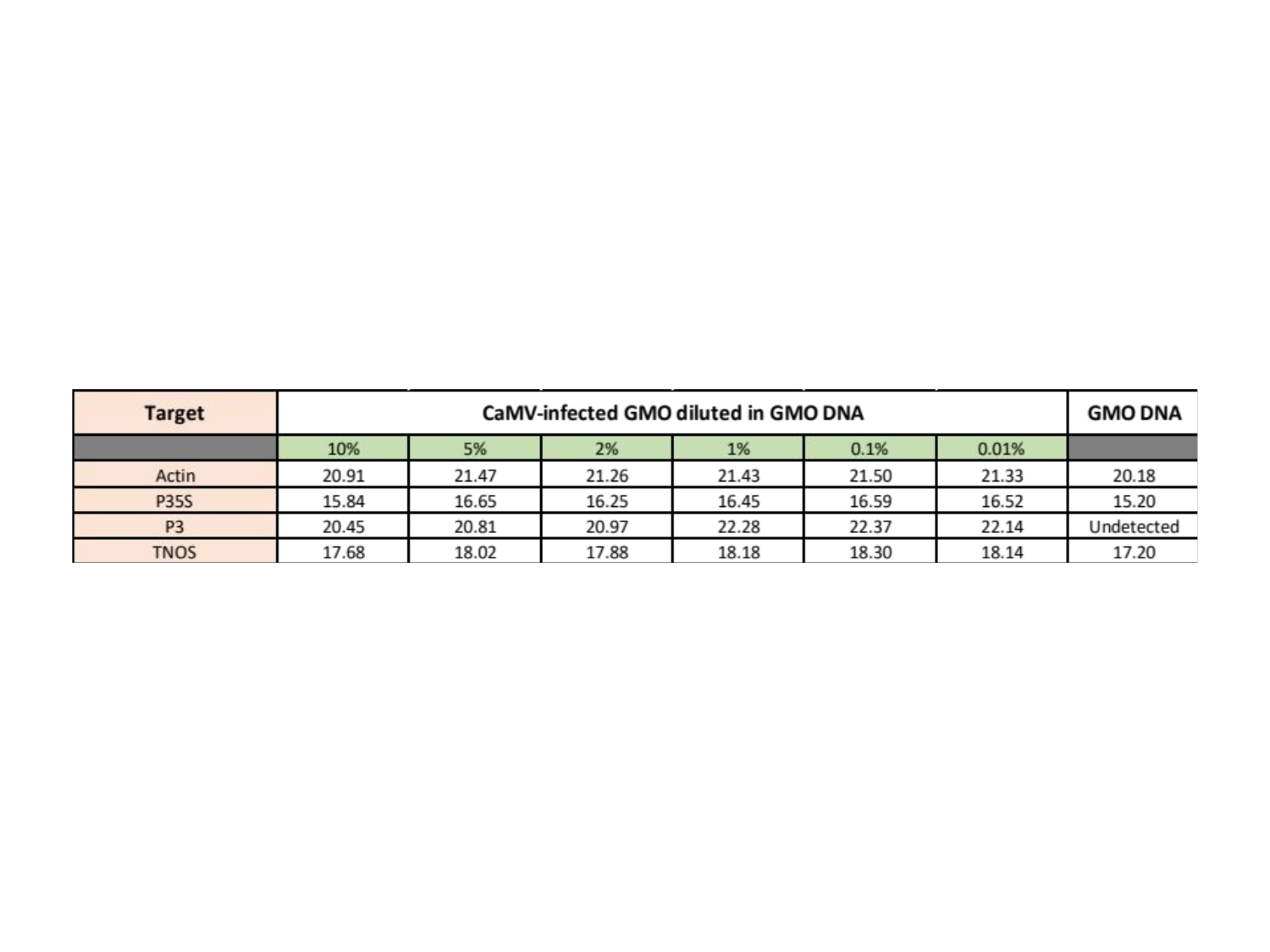

Supplement: Supplementary file 5 — Additional file 5: Table S2. CaMV-infected GM plant DNA versus uninfected GM plant DNA. Ct values for each target (actin, P35S, P3, and TNOS) for different dilution percentages (10, 5, 2, 1, 0.1 and 0.01%) of CaMV-infected GM plant DNA in GM plant DNA, compared to the Ct values obtained from GM plant DNA alone. Here, GMO is short for “GM plant.” [file 12896_2019_571_MOESM5_ESM.pptx]
