# Supplementary material for: Multiplex quantitative PCR for single-reaction genetically modified (GM) plant detection and identification of false-positive GM plants linked to Cauliflower mosaic virus (CaMV) infection
Source: BMC Biotechnol. 2019 Nov 7;19:73. doi: 10.1186/s12896-019-0571-1 (PMC6836441; doi:10.1186/s12896-019-0571-1)
Supplement: Supplementary file 2 — Additional file 2: Figure S1. Primer sets tested by PCR. a Specific primers tested by PCR using the corresponding plasmids as template. After the cloning of the actin and P3 amplification products, two different plasmids (1 and 2) were tested. b Actin primers tested by PCR using plant DNA extract as template (GM Canola, Watercress and Non-GM Canola). c P35S and TNOS specific primers tested by PCR using GM Canola DNA as template. d P3 and TNOS specific primers tested by PCR on CaMV-infected plants (GM Canola, Non-GM Canola and Watercress). [file 12896_2019_571_MOESM2_ESM.pptx]

## Slide 1
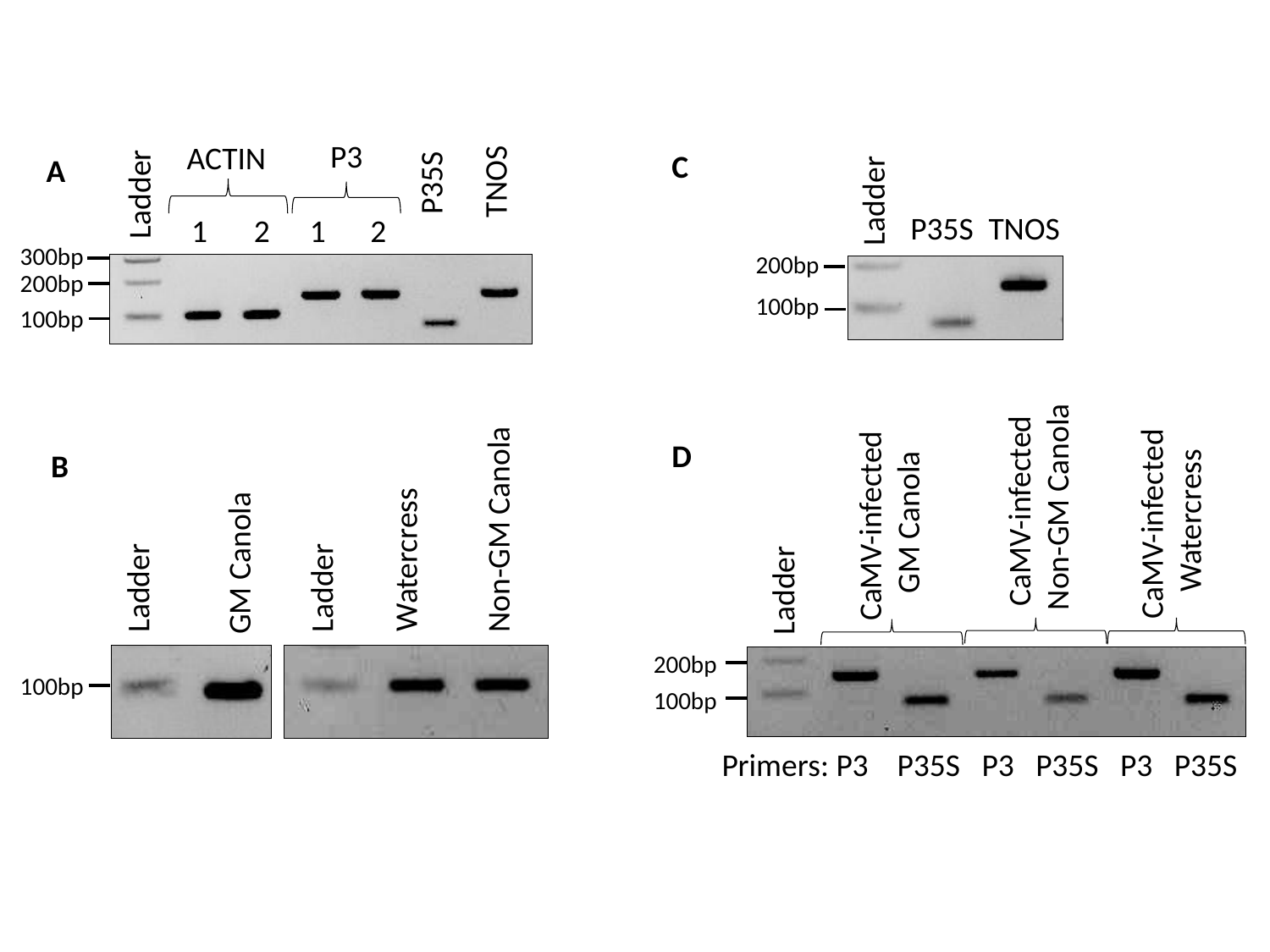

P3
ACTIN
P35S
TNOS
C
A
Ladder
Ladder
P35S
TNOS
1
2
1
2
300bp
200bp
200bp
100bp
100bp
D
B
CaMV-infected
Non-GM Canola
CaMV-infected
Watercress
CaMV-infected
GM Canola
 Non-GM Canola
Watercress
 GM Canola
Ladder
Ladder
Ladder
200bp
100bp
100bp
Primers: P3 P35S P3 P35S P3 P35S
