# Supplementary material for: Multiplex quantitative PCR for single-reaction genetically modified (GM) plant detection and identification of false-positive GM plants linked to Cauliflower mosaic virus (CaMV) infection
Source: BMC Biotechnol. 2019 Nov 7;19:73. doi: 10.1186/s12896-019-0571-1 (PMC6836441; doi:10.1186/s12896-019-0571-1)
Supplement: Supplementary file 3 — Additional file 3: Figure S2. Melting curves for each primer set and probe obtained by SYBR Green assay. Specific primers were tested by SYBR Green qPCR using the corresponding plasmid as template. Melting curves for each set of primers (Forward + Reverse) or using one Primer + the Probe are shown with the temperature on the x-axis and the derivative reporter (△Rn) on the y-axis. The derivative reporter is calculated as the negative first derivative of the normalized fluorescence (Rn) generated by the reporter during PCR amplification. It allows for visualization of the maximum rate of change in fluorescence during the temperature ramp. [file 12896_2019_571_MOESM3_ESM.pptx]

## Slide 1
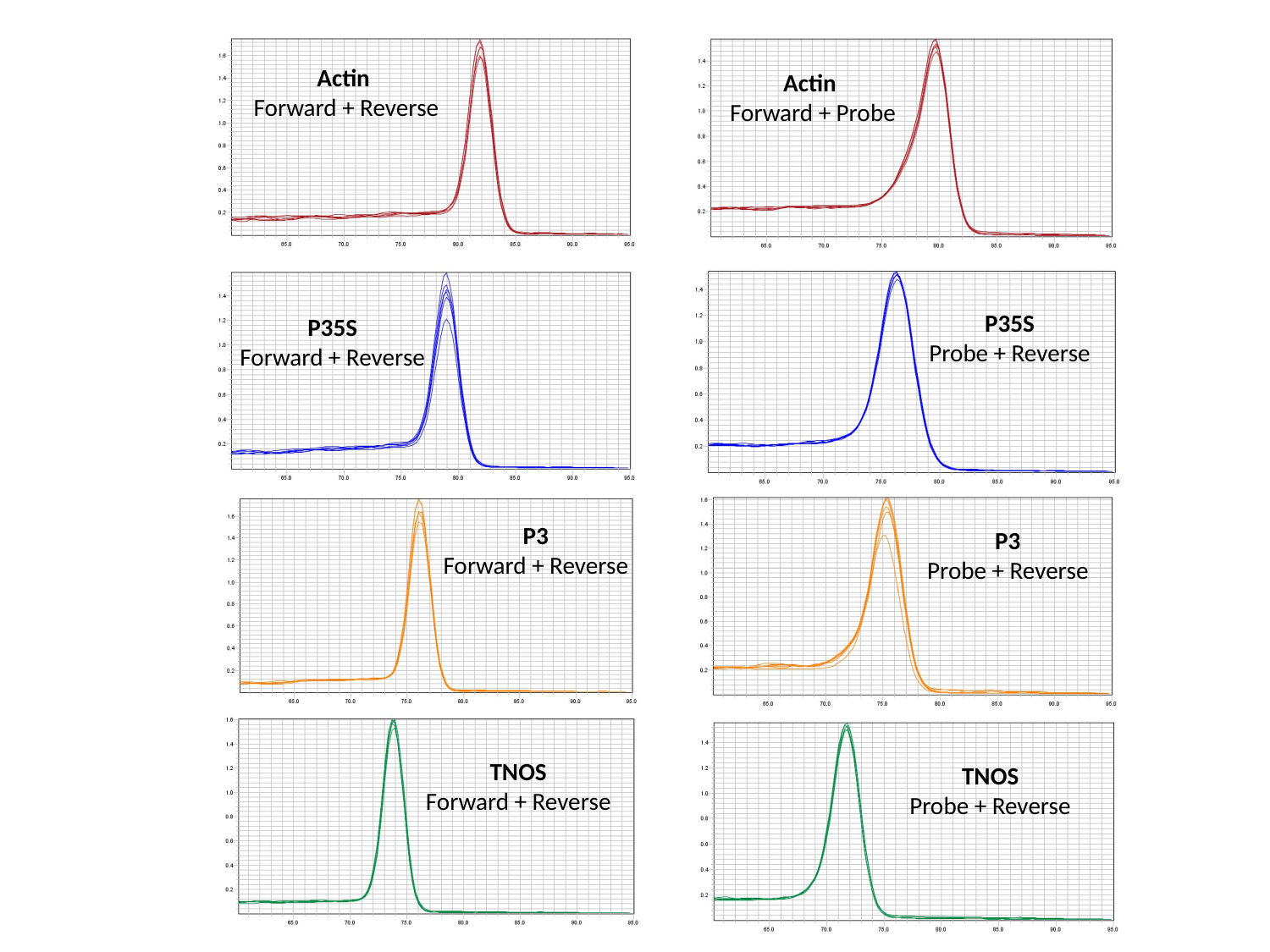

Actin
Forward + Reverse
Actin
Forward + Probe
P35S
Probe + Reverse
P35S
Forward + Reverse
P3
Forward + Reverse
P3
Probe + Reverse
TNOS
Forward + Reverse
TNOS
Probe + Reverse
