# Supplementary material for: Multiplex quantitative PCR for single-reaction genetically modified (GM) plant detection and identification of false-positive GM plants linked to Cauliflower mosaic virus (CaMV) infection
Source: BMC Biotechnol. 2019 Nov 7;19:73. doi: 10.1186/s12896-019-0571-1 (PMC6836441; doi:10.1186/s12896-019-0571-1)
Supplement: Supplementary file 4 — Additional file 4: Figure S3. Standard curves for each target. A serial dilution of the four plasmids mixed together and diluted in water (blue) or in plant DNA (green) for P35S, P3, and TNOS or in bacterial DNA (green) for actin were tested in multiplex qPCR to determine primer efficiency and standard curves for each primer set. [file 12896_2019_571_MOESM4_ESM.pptx]

## Slide 1
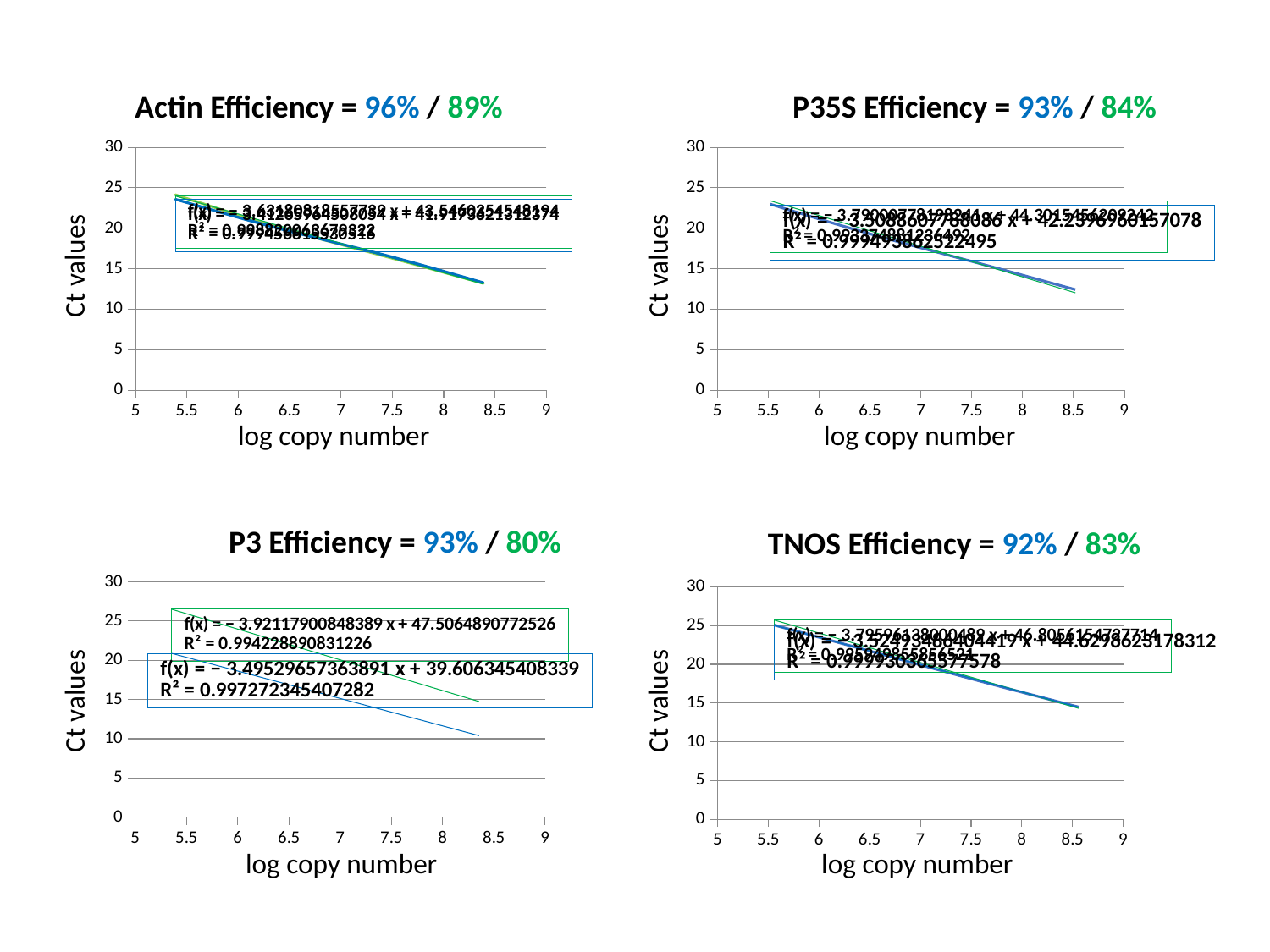

Actin Efficiency = 96% / 89%
P35S Efficiency = 93% / 84%
### Chart
| Category | | |
|---|---|---|
### Chart
| Category | | |
|---|---|---|Ct values
Ct values
log copy number
log copy number
P3 Efficiency = 93% / 80%
TNOS Efficiency = 92% / 83%
### Chart
| Category | | |
|---|---|---|
### Chart
| Category | | |
|---|---|---|Ct values
Ct values
log copy number
log copy number
